# HER2 screening data from ToGA: targeting HER2 in gastric and gastroesophageal junction cancer

**DOI:** 10.1007/s10120-014-0402-y

**Published:** 2014-07-20

**Authors:** Eric Van Cutsem, Yung-Jue Bang, Feng Feng-yi, Jian M. Xu, Keun-Wook Lee, Shun-Chang Jiao, Jorge León Chong, Roberto I. López-Sanchez, Timothy Price, Oleg Gladkov, Oliver Stoss, Julie Hill, Vivian Ng, Michaela Lehle, Marlene Thomas, Astrid Kiermaier, Josef Rüschoff

**Affiliations:** 1 University Hospitals Leuven and KU Leuven, Leuven, Belgium; 2 Seoul National University College of Medicine, Seoul, South Korea; 3 Cancer Institute and Hospital, Beijing, China; 4 Affiliated Hospital (307 Hospital) Cancer Centre, Beijing, China; 5 Seoul National University Bundang Hospital, Seoul National University College of Medicine, Seongnam, South Korea; 6 General Hospital of P.L.A., Beijing, China; 7 Instituto Nacional de Enfermedades Neoplásicas, Lima, Peru; 8 Centro Oncológico Punta Pacífica, Panama City, Panama; 9 The Queen Elizabeth Hospital, Woodville, SA Australia; 10 Regional Oncology Dispensary, Chelyabinsk, Russian Federation; 11 Targos Molecular Pathology GmbH, Kassel, Germany; 12 Roche Products Pty Ltd., Dee Why, NSW Australia; 13 Genentech Inc., South San Francisco, CA USA; 14 F. Hoffmann-La Roche Ltd., Basel, Switzerland; 15 Roche Diagnostics GmbH, Penzberg, Germany; 16 Genentech Inc., Basel, Switzerland; 17 Present Address: McCloud Consulting Group, Gordon, NSW Australia

**Keywords:** Gastric cancer, HER2 testing, Immunohistochemistry, In situ hybridization, Trastuzumab

## Abstract

**Background:**

In the Trastuzumab for GAstric cancer (ToGA) study, trastuzumab plus chemotherapy improved median overall survival by 2.7 months in patients with human epidermal growth factor receptor 2 (HER2)-positive [immunohistochemistry (IHC) 3+/fluorescence in situ hybridization-positive] gastric/gastroesophageal junction cancer compared with chemotherapy alone (hazard ratio 0.74). Post hoc exploratory analyses in patients expressing higher HER2 levels (IHC 2+/fluorescence in situ hybridization-positive or IHC 3+) demonstrated a 4.2-month improvement in median overall survival with trastuzumab (hazard ratio 0.65). The ToGA study provides the largest screening dataset available on HER2 overexpression/amplification in this indication. We further analyzed correlation(s) of HER2 overexpression/amplification with clinical and epidemiological factors.

**Methods:**

HER2-positivity was analyzed by histological subtype, tumor location, geographic region, and specimen type. Exploratory efficacy analyses were performed.

**Results:**

The HER2-positivity rate was 22.1 % across analyzed tumor samples. Rates were similar between European and Asian patients (23.6 % vs. 23.9 %), but higher in intestinal- vs. diffuse-type (31.8 % vs. 6.1 %), and gastroesophageal junction cancer versus gastric tumors (32.2 % vs. 21.4 %). Across all IHC scores, variability in HER2 staining (≤30 % stained cells) was observed in almost 50 % of cases, with increasing rates in lower IHC categories, and did not affect treatment outcome. The polysomy rate was 4 %.

**Conclusions:**

HER2 expression varies by tumor location and type. All patients with advanced gastric or gastroesophageal junction cancer should be tested for HER2 status, preferably using IHC initially. Due to the unique characteristics of gastric cancer, specific testing/scoring guidelines should be adhered to.

**Electronic supplementary material:**

The online version of this article (doi:10.1007/s10120-014-0402-y) contains supplementary material, which is available to authorized users.

## Introduction

Human epidermal growth factor receptor 2 (HER2) is a key driver of tumorigenesis [1], where it is implicated in tumor cell proliferation, apoptosis, adhesion, migration, and differentiation [2]. HER2 has been validated as a prognostic and predictive factor in breast cancer [1, 3], and evidence is growing that HER2 is also a driver of tumorigenesis in gastric and gastroesophageal junction cancer, with studies suggesting that HER2 amplification or overexpression is relatively frequent in this tumor type [2, 4–6]. Studies of HER2-positivity rates in gastric cancer using immunohistochemistry (IHC) and fluorescence or chromogenic in situ hybridization (FISH/CISH) have shown broad variations, ranging from 6.8 % to 34.0 % for IHC [7, 8], 7.1 % to 42.6 % for FISH [7, 9], and 12.2 % to 24.0 % for CISH [6]. Such high variability in HER2-positivity rates can partly be explained by the fact that early reported HER2 data were generated using the breast cancer HER2 testing and/or scoring principles, or were performed with nonvalidated tests. Differences in tumor histology and tumor locations between different cohorts studied may also contribute to the observed high variability of HER2-positivity reported in the literature. Although it remains to be fully established whether HER2 is a valid prognostic factor in gastric cancer, some studies have suggested that HER2-positivity is associated with poor outcomes and more aggressive disease [2, 5, 6, 10], whereas others have shown HER2 to be a favorable prognostic factor [11].

All patients assessed for enrollment into the international, randomized, phase III Trastuzumab for GAstric cancer (ToGA) study were tested for HER2 overexpression and amplification by IHC and FISH, respectively [12]. Patients with advanced gastric or gastroesophageal junction tumors that had HER2 overexpression (IHC 3+) and/or gene amplification (FISH-positive) were eligible for enrollment into the study, if all other inclusion criteria were met, and randomized to trastuzumab (Herceptin^®^, F. Hoffmann-La Roche Ltd., Basel, Switzerland) plus chemotherapy or to chemotherapy alone [12]. Bang and Van Cutsem et al. described the results of this phase III study, showing that trastuzumab in combination with chemotherapy significantly prolonged survival compared with chemotherapy alone in patients with advanced gastric or gastroesophageal junction tumors that showed HER2 overexpression or gene amplification [median overall survival: 13.8 months vs. 11.1 months, respectively; hazard ratio (HR) 0.74; 95 % confidence interval (CI), 0.60–0.91; *P* = 0.0046] [12]. A post hoc exploratory analysis showed a median overall survival of 16.0 months in a subset of patients receiving trastuzumab plus chemotherapy (vs. 11.8 months for patients receiving chemotherapy alone) whose tumors showed higher HER2 protein overexpression (IHC 2+/FISH-positive or IHC 3+), with evidence of a significant interaction test between treatment and the two subgroups of high vs. low HER2 expression (*P* = 0.036 using the log-likelihood ratio test) [12]. These data emphasize the need for high-quality, accurate, and standardized HER2 testing to ensure identification of the patient population that will benefit from trastuzumab.

Here, we present key HER2 screening results from the ToGA study, including data on IHC and FISH testing from the largest population of patients with gastric or gastroesophageal junction tumors to date, and HER2 testing data according to different patient and tumor characteristics. Efficacy data according to HER2 test results are also reported.

## Materials and methods

### Study design and HER2 screening

The study design for ToGA (Clinical trials.gov ID#: NCT01041404: http://clinicaltrials.gov/ct2/show/NCT01041404) has been described previously [12]. Briefly, patients with advanced gastric or gastroesophageal junction cancer displaying HER2 overexpression or amplification were randomized to trastuzumab plus chemotherapy [capecitabine (Xeloda^®^, F. Hoffmann-La Roche Ltd.) or 5-fluorouracil plus cisplatin (XP/FP)] or to chemotherapy alone. All patients provided written informed consent. Investigators performed the human investigations after approval by a Local Human Investigations Committee. HER2 testing was performed in a central laboratory (Targos Molecular Pathology GmbH, Kassel, Germany) by IHC [HercepTest™ (Dako, Glostrup, Denmark)] and FISH [*HER2* FISH pharmDx™ (Dako)] in parallel, and patients whose tumors scored IHC 3+ or had a *HER2*:CEP17 ratio of ≥2.0 by FISH were considered eligible for the study. IHC samples were scored according to the criteria specifically developed for gastric cancer [4], which were applied to the ToGA trial as described by Bang and Van Cutsem et al. [12].

At the screening stage, samples were also classified according to clinical parameters including histological subtype according to the Lauren classification [13] (intestinal, diffuse, or mixed), location of the tumor specimen received (stomach or gastroesophageal junction) and specimen type (biopsy or surgical). Post hoc subgroup analyses for efficacy parameters were performed in two subgroups: patients whose tumors showed lower levels of HER2 protein expression (IHC 0/FISH-positive or IHC 1+/FISH-positive) and those with higher levels of HER2 protein expression [IHC 3+ (including IHC 3+/FISH-negative, IHC 3+/FISH no result, and IHC 3+/FISH-positive) or IHC 2+/FISH-positive].

Efficacy was also assessed in terms of tumor heterogeneity, which was defined as variability in IHC staining in our analysis. We describe two subgroups based on a cutoff of ≤30 % of tumor cells stained vs. >30 % of tumor cells stained [14].

### Statistical analyses

Post hoc exploratory analyses were performed using the statistical program R, Version 1.9.1, and Microsoft Excel. Time-to-event endpoints were analyzed using a Cox regression model. Subgroup analyses were summarized by median values, HRs, and corresponding 95 % CIs. The interaction between treatment and subgroups was assessed using the log-likelihood ratio test. For the analysis of overall tumor response, the proportion of responders in each treatment group (and the two-sided 95 % Pearson-Clopper CIs), together with the odds ratio, is presented.

## Results

### HER2 in advanced gastric and gastroesophageal junction cancer

A total of 3,803 patients were screened for HER2 status; either IHC or FISH testing was successful in 3,665 patients [12]. Results for both IHC and FISH were available from 3,280 patients, and concordance between the two methodologies was calculated with defined categories of IHC-positive (IHC 3+), IHC-negative (IHC 0, IHC 1+, and IHC 2+), FISH-positive, and FISH-negative. Concordance between IHC and FISH with the categories defined above was 87.2 % (2,859/3,280 cases); a total of 354/3,280 samples (10.8 %) were IHC-positive and FISH-positive, and 2,505/3,280 (76.4 %) samples were scored as IHC-negative and FISH-negative (Table 1). A total of 7.5 % (190/2,519) of IHC 0/1+ cases and 74.4 % (566/761) of IHC 2+/3+ cases scored FISH-positive (Table 1). Notably, among patients whose tumors scored IHC 3+, 94.9 % (354/373) were FISH-positive, while 54.6 % (212/388) were FISH-positive in the IHC 2+ group (Table 1). As the IHC 2+ category represents a subgroup of patients with an equivocal result, this subgroup was excluded from further exploratory analyses of concordance. By excluding IHC 2+ samples, concordance between IHC and FISH was 92.8 % (2,683/2,892) (Table 1).

**Table 1:** HER2 testing results from the screening phase of the study, according to immunohistochemistry and fluorescence in situ hybridization

Total screening population^a^ (*N* = 3,280)	IHC 0	IHC 1+	IHC 2+	IHC 3+	Total
FISH-positive	94^b^ (4.9)	96 (15.7)	212 (54.6)	354 (94.9)	756 (23.0)
FISH-negative	1,815^b^ (95.1)	514 (84.3)	176 (45.4)	19 (5.1)	2,524 (77.0)
Total	1,909 (100)	610 (100)	388 (100)	373 (100)	3,280 (100)

Values are expressed as *n* (%)

FISH, fluorescence in situ hybridization; HER2, human epidermal growth factor receptor 2; IHC, immunohistochemistry

^a^With both IHC and FISH results

^b^One patient originally classified as FISH-positive/IHC 0 was reclassified as FISH-negative/IHC 0 after 1 year’s follow-up but was included in the intention-to-treat analyses (HER2-positive)

In total, 810/3,665 (22.1 %) successfully tested patients had IHC 3+ or FISH-positive tumors and were potentially eligible for enrollment (Table 2). Among these 810 patients, 3.1 % (25 patients) had no FISH result available; 1.2 % (10 patients) had no IHC result available; approximately half (398 patients; 49.1 %) had tumors that were IHC 3+, and the remainder were equally distributed between IHC 0/1+ [190 patients; IHC 0 (94 patients) and IHC 1+ (96 patients); 23.5 %] and IHC 2+ (212 patients; 26.2 %; Table 2).

**Table 2:** HER2 testing results in patients potentially eligible for enrollment into ToGA, according to immunohistochemistry and fluorescence in situ hybridization

Patients eligible for ToGA based on HER2 status (*N* = 810)	IHC 0	IHC 1+	IHC 2+	IHC 3+	No IHC
FISH-positive	94^a^ (11.6)	96 (11.9)	212 (26.2)	354 (43.7)	10 (1.2)
FISH-negative	–	–	–	19 (2.3)	–
No FISH result	–	–	–	25 (3.1)	–

Values are expressed as *n* (%)

FISH, fluorescence in situ hybridization; HER2, human epidermal growth factor receptor 2; IHC, immunohistochemistry; ToGA, Trastuzumab for GAstric cancer

^a^One patient originally classified as FISH-positive/IHC 0 was reclassified as FISH-negative/IHC 0 after 1 year’s follow-up but was included in the intention-to-treat analyses (HER2-positive)

HER2 overexpression/gene amplification rates were similar across patients from Europe and Asia (23.6 % vs. 23.9 %; Table 3), but slightly lower in those from Central/South America (16.1 %).

**Table 3:** Proportion of tumors that tested positive for HER2 based on data from all patients screened and from patients successfully screened by immunohistochemistry or fluorescence in situ hybridization, according to patient demographics and tumor characteristics

	All patients^a^	IHC 3+ or FISH-positive	HER2-positivity rate *n/N* ^b^ (%)
All patients screened	3,803	810	n/a
Successfully screened by IHC or FISH	3,665	810	22.1
Specimen location			
Stomach	2,195 (58)	451 (56)	451/2,112 (21.4)
Gastroesophageal junction	208 (5)	65 (8)	65/202 (32.2)
Metastatic sites	322 (8)	51 (6)	51/305 (16.7)
Primary tumor (exact location of specimen not recorded)	1,046 (28)	232 (29)	232/1,017 (22.8)
Not assessed	32 (< 1)	9 (1)	9/29 (31.0)
Tumor subtype			
Diffuse	1,117 (29)	68 (8)	68/1,108 (6.1)
Intestinal	1,916 (50)	606 (75)	606/1,904 (31.8)
Mixed	652 (17)	131 (16)	131/650 (20.0)
Not assessed	118 (3)	5 (<1)	5/118 (4.2)
Specimen type			
Biopsy	2,596 (68)	579 (71)	579/2,492 (23.2)
Surgical	1,199 (32)	231 (29)	231/1,173 (19.7)
Not assessable	8 (<1)	0 (0)	0/0 (0.0)
Region of origin^c^			
Asia–Pacific	1,900 (52)	454 (56)	454/1,900 (23.9)
Europe	795 (22)	188 (23)	188/795 (23.6)
Central/South America	484 (13)	78 (10)	78/484 (16.1)
Other^d^	486 (13)	89 (11)	89/486 (18.3)

Values are expressed as *n* (%)

FISH, fluorescence in situ hybridization; HER2, human epidermal growth factor receptor 2; IHC, immunohistochemistry

^a^As a proportion of the 3,803 patients screened

^b^As a proportion of the 3,665 patients successfully screened according to study protocol, where HER2-positivity was defined as IHC 3+ or FISH-positive [for region of origin, HER2-positivity is calculated based on all patients screened (*N* = 3,803)]

^c^Region-of-origin analysis relates to 809 patients who were IHC 3+ or FISH-positive; one patient was reclassified as FISH-negative/IHC 0 after 1 year’s follow-up but was included in the intention-to-treat analyses (HER2-positive)

^d^Russia and South Africa

Overexpression or amplification of HER2 was more common in patients with intestinal histology compared with those with diffuse histology (31.8 % vs. 6.1 %, respectively; Table 3) and in specimens from the gastroesophageal junction compared with specimens from the stomach (32.2 % vs. 21.4 %, respectively; Table 3). There was also a slightly higher rate of HER2 overexpression/amplification in biopsy specimens compared with surgical specimens (23.2 % vs. 19.7 %, respectively; Table 3).

Variability in HER2 staining intensity (≤30 % positively stained cells) occurred in 294/584 (50.3 %) patients across all IHC categories in all randomized and treated patients. For IHC 3+ samples, 3.1 % (biopsies only), 26.8 %, 31.0 %, and 39.0 % of cases showed HER2 reactivity in <10 %, 10 to 30 %, 31 to 79 %, and 80 to 100 % of tumor cells, respectively. A comparison of the proportion of samples with ≤30 % stained cells per IHC category is shown in Table 4. Variability was greatest in the lower IHC categories. Analysis of the CEP17 signal in tumor samples from evaluable patients showed that polysomy (defined as ≥3 CEP17 signals) occurred in only 23/567 (4.1 %) of cases in patients randomized and enrolled into ToGA with HER2-overexpressing or gene amplification status and in 133/3,317 (4.0 %) of the entire screening population. Twenty-eight out of the 133 cases showed *HER2* gene amplification with a *HER2*:CEP17 ratio of ≥2, and 11 of these cases also demonstrated an IHC 3+ score. The remaining 105 polysomic cases had a FISH ratio <2; only three of these cases were IHC 3+. Seven cases had a gene count of ≥3 and FISH ratio of <2: four scoring IHC 0 and one each scoring IHC 1–3+.

**Table 4:** Proportion of patients with tumor HER2 staining variability (≤30 % stained cells) by IHC category

Patients with ≤30 % stained cells, %	Screening population	Randomized and treated population
IHC 1+	88.5	85.7
IHC 2+	68.5	54.7
IHC 3+	30.5	29.9

HER2, human epidermal growth factor receptor 2; IHC, immunohistochemistry

### Correlation between HER2 expression level and response to trastuzumab

Previously reported findings showed a greater overall survival benefit of trastuzumab plus chemotherapy in the subgroup of patients with higher HER2 expression levels [12]. Trastuzumab plus chemotherapy significantly improved all time-to-event endpoints and the objective response rate vs. chemotherapy alone in this analysis (Table 5).

**Table 5:** Efficacy of trastuzumab plus chemotherapy vs. chemotherapy alone according to HER2 expression status (screened by immunohistochemistry or fluorescence in situ hybridization)

	Lower HER2 expression (IHC 0/FISH-positive or IHC 1+/FISH-positive)		Higher HER2 expression (IHC 2+/FISH-positive or IHC 3+)	
	XP/FP (*n* = 70)	Trastuzumab + XP/FP (*n* = 61)	XP/FP (*n* = 218)	Trastuzumab + XP/FP (*n* = 228)
Overall survival^a^ [12]				
Median (months)	8.7	10.0	11.8	16.0
Hazard ratio	1.07		0.65	
95 % CI	0.70–1.62		0.51–0.83	
Progression-free survival				
Median (months)	4.8	5.3	5.5	7.6
Hazard ratio	1.00		0.64	
95 % CI	0.69–1.45		0.51–0.79	
Time to progression				
Median (months)	5.1	5.5	5.7	7.9
Hazard ratio	1.00		0.64	
95 % CI	0.68–1.47		0.51–0.80	
Duration of response				
Median (months)	4.5	5.4	4.9	7.0
Hazard ratio	0.77		0.5	
95 % CI	0.39–1.52		0.36–0.71	
Tumor response				
Objective response rate, *n* (%)	22 (31.4)	21 (34.4)	77 (35.3)	117 (51.3)
Odds ratio	1.15		1.93	
95 % CI	0.55–2.38		1.32–2.82	

Seven patients did not have an IHC score and were therefore excluded

CI, confidence interval; FISH, fluorescence in situ hybridization; HER2, human epidermal growth factor receptor 2; IHC, immunohistochemistry; ToGA, Trastuzumab for GAstric cancer; XP/FP, capecitabine plus cisplatin or *5*-fluorouracil plus cisplatin

^a^Overall survival in the ToGA population was 11.1 months for chemotherapy alone and 13.8 months for the trastuzumab plus chemotherapy arm (HR 0.74; 95 % CI, 0.60–0.91) [12]

Variability in staining intensity did not adversely affect the overall survival benefit of trastuzumab, although the benefit was numerically lower for patients with tumors exhibiting variable staining, with overlapping CIs (HR 0.84; 95 % CI, 0.63–1.11 vs. HR 0.62; 95 % CI, 0.45–0.86) (please see Online resource 1). When variability in staining intensity was explored by IHC category, patients with IHC 2+ and IHC 3+ scores showed a treatment benefit regardless of variability, with a numerical trend toward greater benefit in patients with >30 % cells stained. In the IHC 2+ category, the HRs were 0.83 (95 % CI, 0.50–1.41) in the ≤30 % subgroup and 0.66 (95 % CI, 0.36–1.18) in the >30 % subgroup. Similarly, in the IHC 3+ subgroup, HRs were 0.71 (95 % CI, 0.40–1.25) and 0.55 (95 % CI, 0.37–0.81) in the ≤30 % and >30 % subgroups, respectively. Again, CIs were wide and overlapping, and the sizes of subgroups compared within the IHC 3+ group were imbalanced (*n* = 86 and 201). Therefore, firm conclusions could not be drawn. Within the IHC 0 and IHC 1+ groups, it was not possible to explore an impact of tumor heterogeneity on treatment benefit as the subgroup with >30 % cells stained was small (*n* = 0 and 10, respectively).

### Correlation between *HER2* amplification level and response to trastuzumab

The *HER2* gene copy number (<6 or ≥6) did not affect overall survival in the subgroups of patients with IHC 0/1+ or IHC 2+ scores, either by stratified or unstratified analyses (*P* > 0.05 in all cases).

## Discussion

Advanced gastric cancer is a difficult-to-treat disease for which there is currently no globally accepted standard of care and for which improved treatments are required [15, 16]. HER2 has been identified as a predictive biomarker in gastric cancer, and efficacy outcomes analyzed in ToGA correlate with the level of HER2 overexpression [12]. Therefore, accurate and standardized HER2 testing is crucial to identify the target population for trastuzumab treatment.

Data from the 3,665 patients who were successfully screened by either IHC or FISH for this study confirm previous reports that HER2 overexpression or amplification is more common in patients with intestinal-type tumors compared with diffuse- or mixed-type tumors [5, 6]. Similarly, our data are consistent with those published elsewhere, suggesting that HER2-positivity rates are higher in specimens from the gastroesophageal junction compared with specimens from the body of the stomach [6, 17]. These differences may be related, as gastroesophageal junction cancers are generally of the intestinal type [18, 19]. These findings suggest that the etiology and pathogenesis of gastroesophageal junction cancer are likely different from those of distal gastric cancer. We also evaluated the relationship between the method of specimen collection (biopsy or surgery) and HER2 overexpression or amplification, and we found that the rates were numerically higher in biopsy samples, although the difference was relatively small (23.2 % vs. 19.7 %, respectively) and unlikely to be clinically relevant. One possible explanation for the higher HER2-positivity rate observed in biopsy specimens compared with surgical specimens could be the different cutoff used to define HER2-positivity by IHC (a cluster of five or more tumor cells for biopsies compared with ≥10 % of tumor cells for surgical specimens). Another could be that too few biopsy samples were collected from patients, as staining variability was fairly common and may have led to inaccurate results.

The difference in HER2-positivity rates between Europe/Asia and Central/South America may have reflected the substantially lower number of patients screened in Central/South America and does not necessarily reflect a different biology.

Among the total screened population with successful IHC or FISH results, 22.1 % displayed HER2 overexpression or amplification (IHC 3+ or FISH-positive and therefore eligible for study entry), while 16.6 % were HER2-positive when applying the definition of higher HER2 overexpression (IHC 2+/FISH-positive or IHC 3+). HER2 screening in the ToGA study was performed in a central testing laboratory (Targos Molecular Pathology GmbH); it is anticipated that HER2-positivity rates may vary when HER2 testing data are reported from regional centers, further highlighting the need for standardized HER2 testing and scoring and the use of accurate and validated assays [14]. Polysomy seems to play a minor role in gastric cancer; in the ToGA study the observed polysomy rate was very low at around 4 %, and very few patients were scored as HER2-negative due to polysomy.

Compared with breast tumors, gastric tumors show higher variability in staining intensities across tissue sections, with focal areas of HER2 overexpression, and common basolateral or lateral membrane staining when evaluated by IHC [4, 12, 14]. Therefore, in the current study, HER2 protein expression levels were assessed according to scoring criteria specific for gastric cancer, which take into account the unique histological features of gastric tumors [4, 12]. The IHC scoring criteria for gastric tumors include not only complete membranous reactivity but also basolateral or lateral membranous reactivity. In addition, the scoring criteria also take into account the variability of staining in this tumor type by not specifying any cutoff for membranous reactivity in biopsy samples; staining in tumor cell clusters of five or more cells is considered positive [14].

In our approach to exploring the impact of variability in staining intensity on overall survival outcomes, we defined variability as ≤30 % positively stained cells [14]. Variability was common, and in the overall analyses it did not adversely impact the overall survival benefit gained from the addition of trastuzumab to chemotherapy (although the benefit was numerically lower for patients with variably stained tumors).

Patients with IHC 2+ and 3+ scores seemed to derive a benefit from trastuzumab, regardless of staining variability. It should be noted that the patient numbers in the analyzed subgroups were small; therefore, the results should be interpreted with caution. In the IHC 0 and 1+ groups, analyses were not possible as the subgroups with >30 % cells stained were too small to assess (*n* = 0 and 10, respectively).

Our report of approximately 50 % variability seems to conflict with previously reported rates of up to one-third [20]; however, in this report we assessed variability in HER2 staining intensity/pattern across all IHC scores in all randomized and treated patients, whereas previous studies assessed variability at the highest staining intensity only. Variability in the IHC 3+ category of ToGA was consistent with the 30 % rate.

Given the frequent occurrence of HER2 staining variability and the slight difference in HER2-positivity rates between biopsy and surgical specimens, it is strongly recommended that a viable number of representative biopsies (ideally 6–8) be collected for HER2 testing, as one or two samples may not give an accurate HER2 result. German guidelines recommend a minimum of eight samples for this reason [21]. Another potential technique for minimizing observer error caused by the variability in staining of gastric tumor material is the use of bright-field HER2 testing methodologies. Bright-field techniques allow for assessment of tumor morphology alongside HER2 evaluation, which permits easier screening of the entire tumor sample and facilitates the identification of focal areas of HER2-positivity [14].

Bang and Van Cutsem et al. showed that the greatest treatment effect was derived in the groups of patients with IHC 2+/FISH-positive and IHC 3+ tumors, while little effect was seen in the IHC 0 and 1+ groups [12]. We further explored cutoffs for *HER2* gene copy number in the IHC 0 and 1+ categories to potentially identify a patient population with a specific *HER2* copy number that may derive a greater benefit from a trastuzumab-based regimen. However, no impact on overall survival was seen when a cutoff of >6 *HER2* gene copies was applied, supporting the initial findings that protein expression shows the strongest association with efficacy and should be the initial testing modality to guide treatment decisions (followed by ISH for equivocal IHC 2+ cases). As sample sizes were low in our assessed sub-subgroup analyses, such findings should be interpreted with caution.

Although *HER2* gene amplification was only assessed using FISH in this study, it is anticipated that bright-field ISH techniques may become the preferred assay, although this will vary regionally depending upon the availability of HER2 testing methodologies. A cohort study including gastric tumor samples from the ToGA trial showed that concordance between silver in situ hybridization (SISH) and FISH was 94.5 %, further suggesting that alternative ISH methods may be a valid testing option for this tumor type [22]. Similarly, concordance studies in breast cancer, for which CISH or SISH are commonly used in place of FISH, have shown concordance rates of 91 % to 100 % between FISH and CISH and 96 % between FISH and SISH [23–26].

Ring studies, where samples are assessed and compared by several laboratories in a sequential manner, can provide valuable information on interlaboratory IHC and ISH consensus and on factors that may lead to discordant results; in breast cancer, ring studies have been proven to be a successful tool for standardizing HER2 testing and scoring, and a similar approach may be effective in gastric cancer [27].

The addition of trastuzumab to chemotherapy significantly prolongs survival compared with chemotherapy alone in patients with advanced gastric or gastroesophageal junction tumors that show HER2 overexpression or gene amplification (median overall survival: 13.8 months vs. 11.1 months, respectively; HR 0.74; 95 % CI, 0.60–0.91) [12]. An exploratory post hoc analysis of efficacy in two distinct patient subgroups, patients with lower levels of HER2 expression (IHC 0/FISH-positive or IHC 1+/FISH-positive) and patients with higher levels of HER2 expression (IHC 3+ or IHC 2+/FISH-positive), showed a significant interaction with trastuzumab treatment (*P* = 0.036). A median overall survival of 16.0 months was achieved with trastuzumab plus chemotherapy in patients with higher HER2 expression levels [12], which compares favorably with previous studies of chemotherapy-only regimens in advanced gastric cancer [28, 29]. The current analysis of the ToGA study also showed that all other secondary efficacy endpoints were improved in response to trastuzumab in the higher HER2 expression subgroup.

In conclusion, these results form the largest set of HER2 testing data from patients with gastric or gastroesophageal junction cancer to date and show that HER2 status varies with tumor location and type. The results also serve to validate HER2 as a predictive biomarker in this disease. In 2010, both the EMA and FDA approved trastuzumab treatment based on the overall survival benefit for patients with metastatic gastric or gastroesophageal junction cancer whose tumors have HER2 overexpression, as determined by an accurate and validated assay. Based on the observed survival benefit, trastuzumab, in combination with a platinum salt and fluoropyrimidine-based chemotherapy, is now considered a treatment option in gastric cancer, and it is recommended that all patients with advanced gastric or gastroesophageal junction cancer should be tested for HER2 expression. Approval and registrations have been obtained in other regions worldwide, and patient eligibility for trastuzumab treatment may vary based on local guidelines. Based on the ToGA findings that the subgroup of patients with higher HER2 protein expression gained the greatest survival benefit [12], it is recommended that all patients with gastric or gastroesophageal junction cancer be tested for HER2 to inform treatment decision-making, preferably using IHC as the initial testing modality. As gastric cancer is a rapidly progressing disease, a multidisciplinary approach to clinical decision-making is required, involving surgeons, oncologists and pathologists. Access to the HER2 status of patients is now vital to assist treatment decision-making, ensuring that patients receive the best possible treatment.

## Electronic supplementary material

Below is the link to the electronic supplementary material.

Supplementary material 1 (DOCX 22 kb)
