# Supplementary material for: HER2 screening data from ToGA: targeting HER2 in gastric and gastroesophageal junction cancer
Source: Gastric Cancer. 2014 Jul 20;18(3):476–84. doi: 10.1007/s10120-014-0402-y (PMC4511072; doi:10.1007/s10120-014-0402-y)
Supplement: Supplementary file 1 — Supplementary material 1 (DOCX 22 kb) [file 10120_2014_402_MOESM1_ESM.docx]

**Online resource 1** Efficacy of trastuzumab plus chemotherapy versus chemotherapy alone according to variability in HER2 staining intensity

|  |  | *XP/FP*  *(*n *= 290)* | | *Trastuzumab + XP/FP*  *(*n *= 294)* | |  |  |
| --- | --- | --- | --- | --- | --- | --- | --- |
| *Percent of stained cells* | *Total* n | n | *Median overall survival, months* | n | *Median overall survival, months* | *HR* | *95 % CI* |
| *All IHC scores* |  |  |  |  |  |  |  |
| 0 % to ≤30 % | 294 | 150 | 10.5 | 144 | 10.9 | 0.84 | 0.63–1.11 |
| >30 % to 100 % | 283 | 138 | 11.8 | 145 | 16.2 | 0.62 | 0.45–0.86 |
| *IHC 0* |  |  |  |  |  |  |  |
| 0 % to ≤30 % | 61 | 38 | 7.2 | 23 | 10.6 | 0.92 | 0.48–1.76 |
| >30 % to 100 % | 0 | 0 | – | 0 | – | – | – |
| *IHC 1+* |  |  |  |  |  |  |  |
| 0 % to ≤30 % | 60 | 26 | 8.8 | 34 | 8.7 | 0.93 | 0.52–1.68 |
| >30 % to 100 % | 10 | 6 | NE | 4 | 7.8 | 3.77 | 0.34–41.64 |
| *IHC 2+* |  |  |  |  |  |  |  |
| 0 % to ≤30 % | 87 | 44 | 11.7 | 43 | 11.4 | 0.83 | 0.50–1.41 |
| >30 % to 100 % | 72 | 35 | 9.2 | 37 | 12.5 | 0.66 | 0.36–1.18 |
| *IHC 3+* |  |  |  |  |  |  |  |
| 0 % to ≤30 % | 86 | 42 | 13.6 | 44 | 18.0 | 0.71 | 0.40–1.25 |
| >30 % to 100 % | 201 | 97 | 12.3 | 104 | 17.9 | 0.55 | 0.37–0.81 |

Seven patients did not have an IHC score and were therefore excluded

CI, confidence interval; HER2, human epidermal growth factor receptor 2; HR, hazard ratio; IHC, immunohistochemistry; XP/FP, capecitabine plus cisplatin or 5-fluorouracil plus cisplatin

**Title: HER2 screening data from ToGA: targeting HER2 in gastric and gastroesophageal junction cancer**

**Journal:** Gastric Cancer

Eric Van Cutsem* **·** Yung-Jue Bang* **·** Feng Feng-yi **·** Jian M. Xu **·** Keun-Wook Lee **·** Shun-Chang Jiao **·** Jorge León Chong **·** Roberto I. López-Sanchez **·** Timothy Price **·** Oleg Gladkov **·** Oliver Stoss **·** Julie Hill^†^ **·** Vivian Ng **·** Michaela Lehle **·** Marlene Thomas **·** Astrid Kiermaier **·** Josef Rüschoff

E. Van Cutsem*

University Hospitals Leuven and KU Leuven, Leuven, Belgium (eric.vancutsem@uzleuven.be)

Y.-J. Bang*

Seoul National University College of Medicine, Seoul, South Korea

F. Feng-yi

Cancer Institute and Hospital, Beijing, China

J. M. Xu

Affiliated Hospital (307 Hospital) Cancer Centre, Beijing, China

K.-W. Lee

Seoul National University Bundang Hospital, Seoul National University College of Medicine, Seongnam, South Korea

S.-C. Jiao

General Hospital of P.L.A., Beijing, China

J. L. Chong

Instituto Nacional de Enfermedades Neoplásicas, Lima, Peru

R. I. López-Sanchez

Centro Oncológico Punta Pacífica, Panama City, Panama

T. Price

The Queen Elizabeth Hospital, Woodville, SA, Australia

O. Gladkov

Regional Oncology Dispensary, Chelyabinsk, Russian Federation

O. Stoss **·** J. Rüschoff

Targos Molecular Pathology GmbH, Kassel, Germany

J. Hill^†^

Roche Products Pty Ltd., Dee Why, NSW, Australia

V. Ng

Genentech Inc., South San Francisco, CA, USA

M. Lehle

F. Hoffmann-La Roche Ltd., Basel, Switzerland

M. Thomas

Roche Diagnostics GmbH, Penzberg, Germany

A. Kiermaier

Genentech Inc., Basel, Switzerland

*These authors contributed equally

^†^Work undertaken while at Roche Products Pty Ltd., Dee Why, NSW, Australia. Current affiliation: McCloud Consulting Group, Gordon, NSW, Australia
